# Profile of patients with hypertensive urgency and emergency presenting to an urban emergency department of a tertiary referral hospital in Tanzania

**DOI:** 10.1186/s12872-018-0895-0

**Published:** 2018-08-02

**Authors:** Patrick J. Shao, Hendry R. Sawe, Brittany L. Murray, Juma A. Mfinanga, Victor Mwafongo, Michael S. Runyon

**Affiliations:** 1 Emergency Medicine Department, Muhimbili National Hospital, Dar es Salaam, Tanzania; 2 Emergency Medicine Department, Muhimbili University of Health and Allied Science, P.O. Box 65001, Dar es Salaam, Tanzania; 3 Department of Emergency Medicine, Carolinas Medical Center, Charlotte, North Carolina USA; 4 Division of Paediatric Emergency Medicine, Emory University School of Medicine, Atlanta, GA USA

**Keywords:** Emergency department, Hypertensive emergency, Hypertensive urgency, And hypertensive crisis, Sub-Saharan Africa

## Abstract

**Background:**

Hypertensive crises are clinical syndromes grouped as hypertensive urgency and emergency, which occur as complications of untreated or inadequately treated hypertension. Emergency departments across the world are the first points of contact for these patients. There is a paucity of data on patients in hypertensive crises presenting to emergency departments in Tanzania. We aimed to describe the profile and outcome of patients with hypertensive crisis presenting to the Emergency Department of Muhimbili National Hospital in Tanzania.

**Methods:**

This was a descriptive cohort study of adult patients aged 18 years and above presenting to the emergency department with hypertensive urgency or emergency over a four-month period. Trained researchers used a structured data sheet to document demographic information, clinical presentation, management and outcome. Descriptive statistics with 95% confidence intervals (CIs) are presented as well as comparisons between the groups with hypertensive urgency vs. emergency.

**Results:**

We screened 8002 patients and enrolled 203 (2.5%). The median age was 55 (interquartile range 45–67 years) and 51.7% were females. Overall 138 (68%) had hypertensive emergency; and 65 (32%) had hypertensive urgency, for an overall rate of 1.7% (95% CI: 1.5 to 2.0%) and 0.81% (95% CI: 0.63 to 1.0%), respectively. Altered mental status was the most common presenting symptom in hypertensive emergency [74 (53.6%)]; low Glasgow Coma Scale was the most common physical finding [61 (44.2%)]; and cerebrovascular accident was the most common final diagnosis [63 (31%)]. One hundred twelve patients with hypertensive emergency (81.2%) were admitted and three died in the emergency department, while 24 patients with hypertensive urgency (36.9%) were admitted and none died in the emergency department. In-hospital mortality rates for hypertensive emergency and urgency were 37 (26.8%) and 2 (3.1%), respectively.

**Conclusion:**

In our cohort of adult patients with elevated blood pressure, hypertensive crisis was associated with substantial morbidity and mortality, with the most vulnerable being those with hypertensive emergency. Further research is required to determine the aetiology, pathophysiology and the most appropriate strategies for prevention and management of hypertensive crisis.

## Background

Hypertension is defined as an elevation of systolic blood pressure (BP) to 140 mmHg or higher or diastolic BP to 90 mmHg or higher. Over 1 billion people in the world have hypertension, 40% of whom are adults older than 25 years [1]. In Africa, it is one of the biggest health concerns, with an estimated prevalence of 46%. Early detection and treatment of hypertension minimize the complications that arise from poorly controlled hypertension, such as heart attack, heart failure, stroke, kidney failure, blindness, and hypertensive crisis, all of which carry significant morbidity and mortality [2].

Hypertensive crises are clinical syndromes that occur as complications of untreated or inadequately treated hypertension [3, 4], and are a frequent reason patients present to health care facilities [5]. Hypertensive emergency is defined as severe hypertension accompanied by acute end organ dysfunction; whereas, hypertensive urgency is defined as severely elevated BP without acute end-organ damage [5]. As such, the categorization of hypertensive emergencies and hypertensive urgencies is based on evidence of acute target organ damage, such as cardiac ischemia, nephropathy, retinopathy, or encephalopathy, rather than on BP level alone [5–7].

In Tanzania, the proportion of patients with hypertensive crisis presenting to acute intake areas remains unknown; however, the overall prevalence of hypertension in Tanzania is estimated to be as high as 40% [8]. Given the prevalence, doctors and other healthcare providers in Tanzania are likely to encounter patients with hypertensive urgencies and emergencies. The prevalence of hypertension is compounded by poverty and lack of proper care for patients presenting in primary health facilities. Most health care facilities lack equipped emergency departments, so the majority of hypertensive patients are treated in clinics and outpatient departments or not treated at all because they remain undiagnosed. The lack of proper facilities and care are huge barriers to early intervention and management [9, 10].

This study examined the prevalence and characteristics of patients with hypertensive urgency and hypertensive emergency seen at the Emergency Department (ED) of Muhimbili National Hospital (MNH). As a tertiary care facility, MNH receives a large number of hypertensive patients, many of whom are experiencing hypertensive crises. The objectives of the study were to determine the rates of hypertensive urgency and emergency among patients presenting to the MNH ED; characterize patient risk factors and clinical presentations; and describe the treatment administered, ED disposition, and overall in-hospital mortality for enrolled patients.

## Methods

### Study design

This was a prospective, descriptive, cohort study of adult patients aged 18 years and above presenting to the ED at MNH with elevated BP from the 1st of September 2015 to the 31st of December 2015.

### Study setting

MNH is the largest National Referral Hospital in Tanzania, which is located in the main commercial city Dar es salaam. The hospital has a bed capacity of 1500 and receives referral from all over the country. The ED is part of the Muhimbili National Hospital and it is the point of entry to the hospital for most patients. The department is relatively new, having been opened in 2010, and is currently staffed by locally trained emergency physicians who oversee the care provided by interns (fresh graduates from medical school), Registrars (registered medical practitioners who are 1 to 3 years post internship), and emergency medicine residents. The ED serves a high-acuity patient population from within Dar es Salaam and receives referral patients from throughout the country, with an estimated annual volume of 50,000 patients and admission rate of 65%. The Tanzanian health system has a mixture of public and private payors. Most patients seen at the ED are in the public category (i.e. the pay a subsidized hospital fee), and few remaining are insured and private. Patients who cannot afford pay for healthcare are subsidized by the government through an exemption process. The top five categories of complaints seen in the department are trauma, infectious, mental health, neoplasm and pregnancy related issues.

### Study protocol

The study inclusion criteria were age ≥ 18 and presentation to the ED with systolic BP of 180 mmHg and higher or diastolic BP of 110 mmHg and higher. Hypertensive *emergencies* included all cases with one or more of the following types of acute end-organ damage: hypertensive encephalopathy; acute pulmonary oedema; congestive heart failure; acute myocardial infarction or unstable angina pectoris; and progressive renal insufficiency or significantly reduced urine output. Hypertensive urgency was defined as elevated blood pressure meeting inclusion criteria, but without evidence of acute end organ dysfunction. The conditions were diagnosed clinically and by diagnostic tests such as blood chemistry for serum creatinine and Urea, 12-lead electrocardiography, computed tomography, and ultrasound imaging as appropriate. In the absence of end-organ damage, all other hypertensive crises were considered by exclusion to be hypertensive urgencies.

Study personnel were available to screen and enroll consecutive patients each Monday, Wednesday, Friday, and Saturday during the study period. Screening was for 24 h, beginning at 0800 h each of these days, ensuring that enrollment spanned, day, evening, night, and weekend hours. For all those who were eligible, and consented to the study, trained researchers completed a structured data sheet documenting demographic information, clinical presentation, diagnostic evaluations, EMD treatment, outcome and disposition. Patients were followed in the wards to collect information on subsequent treatment and disposition. For patients discharged from the ED, follow-up was conducted via telephone.

### Measurements

Vital signs (including blood pressure) of consecutive patients presenting the ED were measured by using a digital vital signs monitor (Philips sure sign VS2+) that is available for routine care in the triage area of the ED. Patients found to be hypertensive on initial screening underwent manual blood pressure confirmation (YUYUE XB-11 aneroid sphygmomanometer GB3053–93). Blood pressure measurements were preferentially completed in the sitting position (for the majority of patients) and in the supine position for those patients who were unable to sit.

### Key outcome measures

The primary outcomes were the rates of hypertensive urgency and emergency. Secondary measures included: patient risk factors and clinical presentations, treatment provided, patient disposition, and in-hospital mortality.

### Data analysis

Data were entered into an Excel spread sheet (Microsoft Corporation, Redmond, WA, USA) and analysed with StatsDirect version 3.0.133 (StatsDirect Ltd., Cheshire, UK). Descriptive statistics, including counts (percentages), means (standard deviations), medians (interquartile ranges [IQR]), and 95% confidence intervals (CIs) are reported as appropriate. Comparisons between groups were performed using the Chi Square or Fisher’s exact test for proportions and Student’s T-test or the Mann-Whitney U test for continuous variables, as appropriate to the distribution of the data. Two-sided *p*-values < 0.05 were considered significant. The rates of hypertensive urgency and emergency were calculated by dividing patients diagnosed with each condition by the total number of patients who visited the ED during the screening periods. In-hospital mortality was calculated by dividing the number of patients who died by the total number of patients enrolled.

## Results

### Demographic characteristics

**Fig. 1:**
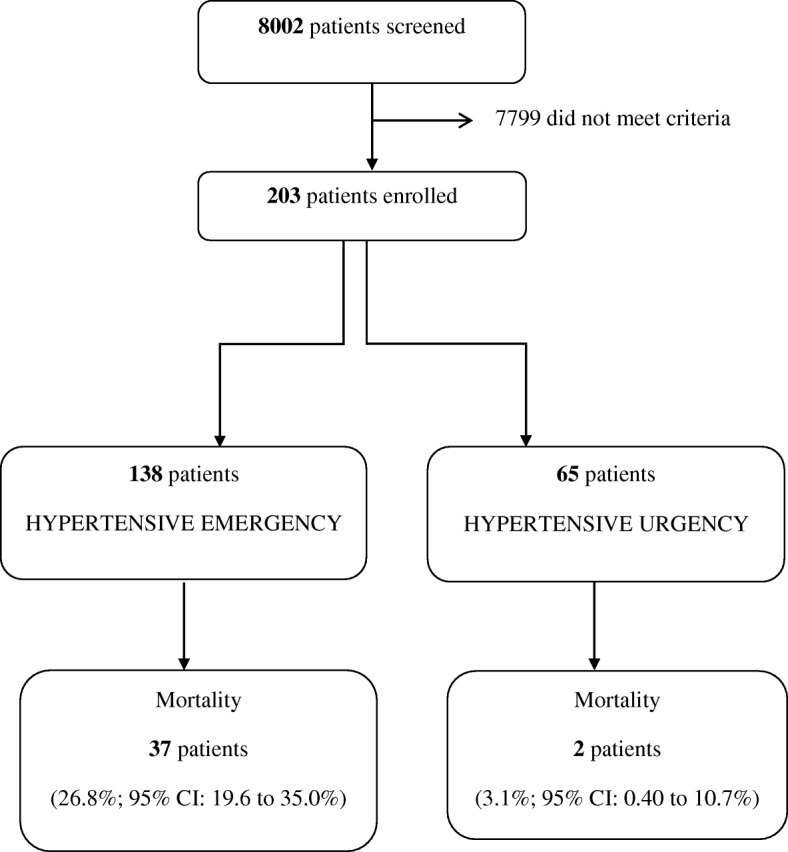
The study flow chart showing the enrolment of patients with hypertensive crisis

A total of 8002 patients presented to the ED during the screening periods over the four-month study (as shown in Fig. 1). Of the 203 patients enrolled, 138 patients (68.0%) had hypertensive emergency and 65 had hypertensive urgency. As such, the rates of hypertensive emergency and urgency among patients seen in our ED during these times were 1.7% (95% CI: 1.5 to 2.0%) and 0.81% (95% CI: 0.63 to 1.0%), respectively.

The median age of enrolled patients was 55 [interquartile range (IQR) 45–67], and the majority were female, married, had a primary school education, and were unemployed. Patient characteristics are displayed in Table 1.

**Table 1:** Patient characteristics^a^

	Overall *N* = 203	Emergency *n* = 138	Urgency *n* = 65	*p*-value
Sex				
Male	95 (46.8)	62 (44.9)	33 (50.8)	0.44
Female	108 (53.2)	76 (55.1)	32 (49.2)	
Age				
Median (IQR) years	55 (45–67)	54 (43–67)	60 (50–67)	0.25
Marital status				
Single	17 (8.4)	9 (6.5)	8 (12.3)	0.28
Married	146 (71.9)	98 (71.0)	48 (73.8)	
Divorced	14 (6.9)	10 (7.3)	4 (6.2)	
Widow	26 (12.8)	21 (15.2)	5 (7.7)	
Level of Education				
University	21 (10.3)	14 (10.2)	7 (10.8)	0.43
Advanced level (High School)	3 (1.5)	1 (0.7)	2 (3.1)	
Ordinary level (Secondary School)	45 (22.2)	34 (24.6)	11(16.9)	
Primary	111 (54.7)	74 (53.6)	37 (56.9)	
None	13 (6.4)	10 (7.3)	3 (4.6)	
Others	10 (4.9)	5 (3.6)	5 (7.7)	
Employment				
Government	31 (15.3)	24 (17.4)	7 (10.8)	0.49
Private	17 (8.4)	13 (9.4)	4 (6.1)	
Self	60 (29.5)	39 (28.3)	21 (32.3)	
Unemployed	95 (46.8)	62 (44.9)	33 (50.8)	

^a^Values and counts (%) unless otherwise specified

### Patient risk factors

Risk factors for hypertensive urgency and emergency are shown in Table 2. About one- fifth of enrolled patients were current alcohol users, and over a quarter reported a past history of alcohol use. Less than 10% were current cigarette smokers. Almost 80% of patients reported they did not engage in physical exercise. A history of hypertension was reported by 162 patients (80.2%). Of these 162 patients, nearly half were not on regular medications, and about two-thirds were not on a regular clinic visit schedule. The overall mean systolic blood pressure at presentation to EMD was 188; other clinical values (data not shown) include a mean heart rate of 93, respiratory rate of 23, and peripheral oxygen saturation (SpO_2_) of 98% on room air.

**Table 2:** Risk factors

	Overall (%) N = 203	Emergency (%) n = 138	Urgency (%) n = 65	*p*-value
Alcohol *n* = 202				
Current alcohol use	44 (21.8)	33 (24.1)	11 (16.9)	0.26
Past alcohol use	54 (26.7)	33 (24.1)	21 (32.3)	0.21
Cigarette n = 202				
Current cigarette use	15 (7.4)	12 (8.8)	3 (4.6)	0.32
Past cigarette use	54 (26.7)	33 (24.1)	21 (32.3)	0.21
Exercises *n* = 200				
Performing exercise	41 (20.5)	25 (18.5)	16 (24.6)	0.28
Types of exercises				
Jumping	14 (34.1)	6 (24)	8 (50)	0.43
Walking	12 (29.3)	7 (28)	5 (31.2)	
Frequency of exercises				
Daily	16 (39)	9 (36)	7 (43.8)	0.81
2 to 3 times per week	12 (29.3)	8 (32)	4 (25)	
Once per week	9 (22)	6 (24)	3 (18.8)	
Hypertension n = 202				
SBP at presentation (mean)	188 (SD 27)	190 (SD 30)	184 (SD 19)	0.08
Hypertension history	162 (80.2)	114 (83.2)	48 (73.8)	0.0002
Regular medication use	82 (59)	60 (58.8)	22 (59.5)	0.19
Regular clinic visit	55 (36.4)	39 (36.4)	16 (36.4)	0.59
Stop medication	67 (56.3)	51 (60.0)	16 (47.1)	0.81

*SBP* systolic blood pressure; *SD* standard deviation

### Clinical presentation and outcomes

Altered mental status and headache were the two most common clinical presentations in the hypertensive emergency group, while body weakness and abdominal pain were the most common symptoms in the hypertensive urgency group. Most patients with hypertensive urgency were discharged (63.1%), while only 16.7% of patients with hypertensive emergency were discharged (*p* < 0.0001). As shown in Tables 3, 111 (80.4%) patients with hypertensive emergency were admitted to the general medical ward as compared to 24 (36.9%) of those with Hypertensive urgency (*p* < 0.0001). One patient with hypertensive emergency was admitted to the ICU, and three (2.2%) died in the ED. No patient with hypertensive urgency died in the ED. Overall in-hospital mortality rates for patients with hypertensive emergency and urgency were 26.8 and 3.1%, respectively (*p* < 0.0001). Overall, hypertension 76 (37.4%), cerebral vascular accident 63 (31.0%), and Renal failure 25 (12.3%) were the top three most frequent final diagnoses Table 4.

**Table 3:** Clinical presentation and outcome

	Overall (%) N = 203	Emergency (%) n = 138	Urgency (%) n = 65	
Presenting complaints				
AMS	74 (36.5%)	74 (53.6%)	0	
Headache	71 (35%)	71 (51.4%)	0	
Chest pain	26 (12.9%)	26 (18.8%)	0	
Blurred vision^a^	21 (10.3%)	20 (14.5%)	1 (1.5%)	
Decreased urine output	11 (5.4%)	11 (8%)	0	
Shortness of Breath^b^	47 (23.2%)	46 (33.3%)	1 (1.5%)	
GCS ≤14	61 (30%)	61 (44.2%)	0	
EMD Outcome				p-value
Admitted to general ward	135 (66.5%)	111 (80.4%)	24 (36.9%)	< 0.0001
Admitted to ICU	1 (0.5%)	1 (0.7%)	0	
Discharged Home	64 (31.5%)	23 (16.7%)	41 (63.1%)	< 0.0001
Died at EMD	3 (1.5%)	3 (2.2%)	0	
Overall mortality				
EMD plus in-hospital	39 (19.2%)	37 (26.8%)	2 (3.1%)	< 0.0001

^a^The patient in the urgency group had EM final diagnosis of refractive error

^b^patient in the urgency group had an EM final diagnosis of severe pneumonia

**Table 4:** Final patient diagnosis

	Overall	Emergency	Urgency
	N = 203	N = 138	N = 65
Hypertension	76 (37.4%)	56 (40.6%)	20 (30.8%)
Cerebral Vascular Accident	63 (31.0%)	63 (45.7%)	0
Renal failure	25 (12.3%)	21 (15.2%)	4 6.2%)
Hypertensive emergency	20 (9.9%)	20 (14.5%)	0
Diabetic Mellitus	20 (9.9%)	16 (11.6%)	4 (6.2%)
Heart failure	15 (7.4%)	11 (8.0%)	4 (6.2%)
Pulmonary oedema	11 (5.4%)	11 (8.0%)	0
Hypertensive urgency	9 (4.4%)	6 (4.3%)	3 (4.6%)
Myocardia Ischemia	6 (3.0%)	6 (4.3%)	0

### ED management

Table 5 provides detail of treatment administered in the ED. Intravenous (IV) antihypertensive medication was administered to 41 patients with hypertensive emergency (29.7%), while 84 (60.9%) of these patients received no antihypertensive medication. Seven of the 65 patients with hypertensive urgency (10.8%) were given an IV antihypertensive. The IV antihypertensives commonly used were labetalol, nitroglycerine, and hydralazine. The sublingual antihypertensive used was nitroglycerin.

**Table 5:** Medication and treatment administered in the emergency department

	Overall	Emergency	Urgency	*p*-value
Antihypertensive	N = 203	N = 138	N = 65	0.76
Oral	3 (1.5%)	3 (2.2%)	0	
Sublingual^a^	12 (5.9%)	10 (7.2%)	2 (3.1%)	
IV	48 (23.6%)	41 (29.7%)	7 (10.8%)	
Morphine	10 (4.9%)	7 (5.1%)	3 (4.6%)	0.92
Aspirin	2 (1.0%)	2 (1.4%)	0	N/A
Antibiotics	40 (19.7%)	31 (22.5%)	9 (13.8%)	0.15
Fluids	37 (18.2%)	25 (18.1%)	12 (18.5%)	0.95
Others	38 (18.7%)	29 (21.0%)	9 (13.8%)	0.22

^a^The sublingual medication used was nitroglycerin

## Discussion

The diagnosis and management of hypertensive crisis poses a unique challenge, especially in low-income countries like Tanzania [11]. To the best of our knowledge, this is the first descriptive study reporting the prevalence of hypertensive crisis in an ED population in Tanzania. The prevalence is similar to what has been reported in most sub-Saharan Africa countries, which ranges between 0.5 and 4.0% [12].

Our study population was similar to the overall patient population in Tanzania’s health care system, which is commonly affected by ischemic heart disease and cerebrovascular disease [1]. Most patients in our study self-reported risk factors for cardiovascular disease (example. Cigarette smoking, lack of physical exercise, sedentary work), along with poor compliance with antihypertensive medications, which has been associated with hypertensive emergency and urgency. These findings are similar to observations made in other sub-Saharan countries [12–14], in which obesity, history of hypertension, low socioeconomic status, poor health literacy, and lack of compliance with drug treatment were mentioned as factors associated with hypertensive emergency and urgency.

The clinical presentation of hypertensive crisis varies widely depending on the underlying pathology [15, 16]. We found that nearly half of patients with hypertensive emergency presented with altered mental status or headache. Conversely, patients with hypertensive urgency presented mostly with generalized body weakness and abdominal pain. These symptoms could indicate a wide range of possible diagnoses and highlight the need for a thorough evaluation prior to disposition.

In this study, 61 (44.2%) patients with hypertensive emergency had a GCS < 14, which was the most common physical finding, followed by focal neurological deficit (31.9%) and crepitations (11.6%). These findings are similar to other studies in sub-Saharan Africa and Europe [12, 17, 18]. For those with hypertensive urgency, lower limb swelling and pallor were among the most common physical findings identified.

Most patients in our study did not receive recommended ED management of their hypertension. The use of intravenous antihypertensive medication in acute management of hypertensive emergency is recommended as standard treatment [3, 7, 19], while oral antihypertensives and appropriate investigation and follow-up are recommended for patients with hypertensive urgency [19]. In this study, nearly two-thirds of patients with ED diagnosis of hypertensive emergency did not receive any antihypertensive. We think this may be due to both a lack of available intravenous medications and provider hesitancy to rapidly lower blood pressure. Ironically, we found that seven patients with urgency were given intravenous antihypertensive medications and two were given sublingual nitroglycerine. Yet, the departmental protocol clearly limits use of intravenous and sublingual antihypertensive medications to patients with hypertensive emergency. These findings indicate the need for further study to evaluate provider compliance with hypertensive crisis protocols since the MNH emergency department has had such protocols available for over 3 years prior to this study.

Another concerning finding was that 23 (16.7%) patients with hypertensive emergency were discharged from the ED. Nearly all of these patients received a cardiac consultation while in the ED, but the standard consultative discussion on benefits of admission versus discharge was not documented. Most of these discharged patients were given oral medications and asked to follow-up in cardiac clinic. These findings reveal a need to re-evaluate existing interdepartmental standard operating procedures. A follow-up study should also be performed to assess the impact of the current practice on optimization of patient care and outcomes.

CVA was the top ED diagnosis, followed by diabetes mellitus, renal failure, heart failure, pulmonary oedema, and myocardial ischemia. Only one patient with hypertensive emergency was admitted to the ICU. This observation indicates a very low rate, which is contrary to international guidelines that recommend admission to ICU and/or a high dependent unit (HDU) for patients with hypertensive emergency [3, 19, 20]. Studies have shown improved outcomes for these patient populations when treated in the ICU setting [3]. Unfortunately, there is a significant shortage of ICU facilities at MNH as reported previously [21, 22]. During the time of this study, there were only five ICU beds available out of 1500 total hospital beds. As such, many patients who would be more ideally cared for in the ICU setting were instead admitted to a medical ward.

In-hospital mortality among patients with hypertensive emergency was 26.8%. While factors contributing to the high mortality might include severity of illness and existing comorbidities, the most important factor was likely the inability to provide advanced care to critically ill patients due to lack of resources [22]. Only two patients with hypertensive urgency (3.1%) died in hospital, and these patients had severe comorbidities (i.e. sepsis, hypokalaemia, and bladder tumour).

## Limitations

The study was limited by the fact that it was performed in a national, public, tertiary hospital, where most patients are public- rather than private-pay patients, thereby limiting the generalizability of our findings. Likewise, the lack of ICU facilities makes our outcome results not generalizable to centres with good ICU care. In addition, available treatment was occasionally restricted due to resource limitations; hence, the observed treatment strategies may be due not to physician preference, but rather medication availability. Finally, we were not able to screen all patients who presented to the ED during the study period due to research personnel limitations. Instead, we chose to enroll consecutive patients presenting during one of four 24-h periods each week. As our screening and enrolment spanned day, evening, night, and weekend hours, we believe our sample is likely representative of the overall patient population presenting to the MNH ED.

## Conclusion

In our cohort of adult patients with elevated blood pressure, hypertensive crisis was associated with substantial morbidity and mortality, with the most vulnerable being those with hypertensive emergency. Further research is required to determine the aetiology, pathophysiology and the most appropriate strategies for prevention and management of hypertensive crisis.

## Abbreviations

AMS: Altered Mental Status
BP: Blood Pressure
CT-Scan: Computerized Tomography Scans
CVA: Cerebral vascular Accident
EM: Emergency Medicine
EMD: Emergency Medicine Department
ICU: Intensive Care Unit
IV: Intravenous
JNC: Joint National Committee
MNH: Muhimbili National hospital
MUHAS: Muhimbili University of Health and Allied Sciences
